# Supplementary material for: WordCommentsAnalyzer: A windows software tool for qualitative research
Source: F1000Res. 2018 Sep 4;7:536. Originally published 2018 May 3. [Version 2] doi: 10.12688/f1000research.14819.2 (PMC6137411; doi:10.12688/f1000research.14819.2)
Supplement: Supplementary file 3 [file f1000research-7-17614-s0002.tgz › b2a79496-4ada-47c4-9682-a99ce867836d.zip!/Eight Iranian Health Journals/Journal of Health System Research _46.docx]

© Journal of Health System Research . This work is licensed under CC BY-NC 4.0 (https://creativecommons.org/licenses/by-nc/4.0/).

Volume & Issue: دوره 6، شماره 3؛ 1389

Title: بررسی نشت فلزات سنگين از لوله‌های پی وی سی و پلی پروپیلن مصرفي در شبكه آبرساني شهر اصفهان

http://hsr.mui.ac.ir/index.php/jhsr/article/view/90

مقدمه: در توليد مواد پليمري مقدار زيادي مواد شيميايي استفاده مي‌گردد كه اين مواد در طول زمان از مواد پليمري نشت كرده و مي‌توانند سبب بروز مشكلات بهداشتي گردند. روش‌ها: در اين تحقيق چهار نوع لوله پلی‌پروپیلن (PP) و دو نوع لوله پی‌وی‌سی (PVC) مورد آزمايش قرار گرفت و غلظت و ميزان نشت فلزات سنگين (سرب، كادميوم، نيكل، كروم، روي و قلع) در دماي 20 درجه سانتی‌گراد مطالعه و با استاندارد ملي ايران و سازمان حفاظت محیط زیست آمریکا (EPA) و رهنمودهاي سازمان جهانی بهداشت (WHO) مقايسه گرديد. يافته‌ها: ميانگين ميزان سرب در لوله‌هاي PVC نو و كهنه نسبت به ساير لوله‌ها بسيار بيشتر بوده و بين مقادير سرب در اين دو لوله نسبت به ساير لوله‌ها از نظر آماري تفاوت معني‌داري وجود دارد. همچنين ميزان نشت اين فلز در آب از لوله PP توليدي كارخانه (الف) و (ب) بيشتر است. در اين دما، ميزان نشت سرب از لوله‌ها از استاندارد ملي كم‌تر، ولي در لوله PP توليدي كارخانه (الف) و (ب) از استاندارد EPA و رهنمودهاي WHO بیش‌تر بود. در مورد كادميوم نيز ميزان نشت لوله PP توليدي كارخانه (پ) از هر سه استاندارد و رهنمود بیش‌تر بود. نتيجه‌گيري: با توجه به ارتباط مستقيم استفاده از لوله‌هاي پلي‌مري با سلامتي مصرف‌کنندگان، ضمن ضرورت کنترل جدي توليد لوله‌هاي مزبور از نظر بهداشتي توسط مراجع ذيربط، تغيير نوع تثبيت‌كننده و عدم استفاده از رنگ دانه‌هاي با پايه فلزات سنگين پيشنهاد می‌شود.

Title: بررسي تأثير پوستر‌‌هاي آموزشي بر ارتقای سطح آگاهی و نگرش ساکنان آپارتمان‌های منتخب شهر اصفهان در زمينه بهداشت دهان و دندان

http://hsr.mui.ac.ir/index.php/jhsr/article/view/60

مقدمه: امروزه بهداشت دهان و دندان يكي از معضلات بهداشتي كشور مي‌باشد. پوسترهای آموزشی، چنانچه به صورت مناسب طراحی و به کار گرفته شوند، یکی از راه‌های ساده و مقرون به صرفه آموزش بهداشت می‌باشد. هدف از انجام اين پژوهش، بررسي تأثير آموزش بهداشت دهان و دندان از طريق نصب پوستر آموزشي در آسانسور و تابلو اعلانات آپارتمان‌‌ها بود. روش‌ها: پژوهش حاضر به صورت نيمه تجربي با نمونه‌گيري از افراد ساکن در 10 مجموعه آپارتمان آسانسوردار نقاط مختلف شهر اصفهان در سال 1387 انجام شد (5 آپارتمان به عنوان مورد و 5 آپارتمان به عنوان شاهد انتخاب شدند). در مجموع 126 نفر در این مطالعه شرکت کردند. اطلاعات مربوط به آگاهی و نگرش در زمینه بهداشت دهان و دندان، با پرسش‌نامه خود ايفا در دو مرحله قبل و بعد از نصب پوستر‌‌هاي آموزشي جمع‌آوري گردید. در آپارتمان‌های مورد، از پوستر آموزشي بهداشت دهان و دندان استفاده گرديد. پس از يك ماه پوستر‌‌ها جمع‌آوري گرديد. پس از دو ماه مجدداً پرسش‌نامه‌ها در ميان تمام افراد (گروه مورد و شاهد) توزيع گرديد. اطلاعات به روش آمار تحليلي با نرم‌افزار SPSS مورد تجزيه وتحليل قرار گرفت. یافته‌ها: 5/62 درصد از افراد زن و 5/37 درصد مرد بودند.43 درصد از آنان مدرك تحصيلي ديپلم داشتند، 8/25 درصد از نظر نمره آگاهي و 7/86 درصد از نظر نمره نگرش وضعيت نامطلوب داشتند. قبل از مداخله وضعيت آگاهي، نگرش و عملكرد افراد دو گروه تفاوت معني‌داري نداشت و پس از نصب پوسترهاي آموزشي وضعيت آگاهي، نگرش و عملكرد افراد گروه مورد اندكي افزايش يافت اما اين افزايش از نظر آماري معني‌دار نبود. نتیجه‌گیری: روش آموزشي به كار رفته در اين پژوهش كارآيي لازم جهت ارتقا سطح آگاهي، نگرش وعملكرد افراد را نداشت. پيشنهاد مي‌شود جهت تكميل اثر بخشي پوستر‌‌ها از ساير روش‌‌هاي آموزش بهداشت نیز استفاده شود. واژه‌‌های كليدی: پوستر آموزشي، آگاهي، نگرش، بهداشت دهان و دندان، آسانسور.

Title: اکسيداسيون 4- کلروفنل با کاربرد توأم فن آوری اکسيداسيون پيشرفته و مايکروويو اصلاح‌شده در فاضلاب صنايع پتروشيمی و شيميایی

http://hsr.mui.ac.ir/index.php/jhsr/article/view/111

مقدمه: 4- کلروفنل يکی از آلاينده‌های رايج در فاضلاب توليدی از صنايع شيميایی و پتروشيمی است. اين ماده به دليل حضور کلر و حلقه بنزنی در آن، در برابر تجزيه بيولوژيکی مقاوم است. روش‌های متعددی جهت حذف اين آلاينده در محيط، مورد استفاده قرار گرفته است که در اين ميان روش‌های اکسيداسيون پيشرفته دارای قابليت بيشتری می‌باشند. روش‌ها: در اين مطالعه امکان حذف اين آلاينده با استفاده از يک دستگاه مايکروويو خانگی اصلاح‌شده به تنهایی و همراه با افزودن ماده اکسيد‌کننده (پراکسيد هيدروژن)، مورد بررسی قرار گرفت. همچنین تأثير pH، زمان تماس، قدرت امواج مايکروويو و غلظت اوليه ماده آلی مورد بررسی قرار گرفت. یافته‌ها: کارایی حذف اين ماده شيميایی به غلظت پراکسيد هيدروژن بستگی دارد به طوري که غلظت بهينه ماده اکسيدکننده 1/0 مولار می‌باشد و با افزايش آن از مقدار بهینه ميزان حذف کاهش می‌یابد. نتايج اين مطالعه در خصوص تأثير pH نشان می‌دهند که در 5/10pH =  حداکثر کارایی حذف حاصل می‌شود. همچنين ميزان تجزيه اين ماده به شدت به غلظت اوليه آن بستگی دارد. توان مايکروويو عامل مهمی در حذف اين آلاينده می‌باشد و با افزايش انرژی خروجی، ميزان حذف افزايش می‌یابد. مقدار انرژی الکتريکی مورد استفاده در اين روش 17460 کيلووات ساعت به ازای هر کيلوگرم ماده آلی تجزيه شده می‌باشد. نتیجه‌گیری: بررسی نتايج اين مطالعه در خصوص حذف 4- کلروفنل با مايکروويو اصلاح‌شده حاکی از بالا بودن راندمان حذف اين ماده آلی با استفاده از مايکروويو در محيط قليایی و امواج بالای اشعه مایکروویو می‌باشد. با اين حال مصرف برق بالا توسط اين روش می‌بايست مورد توجه قرار گيرد.

Title: بررسي دانش دانشجويان دانشگاه‌هاي علوم پزشكي غرب كشور در محيط‌هاي web 2.0

http://hsr.mui.ac.ir/index.php/jhsr/article/view/128

مقدمه: فناوري‌ وب 2، رويكرد جديد در فرآيند اطلاع‌رساني و اشتراك‌داده‌ها در اينترنت است. مشخصه بارز تمركز بر تعامل كاربران و توسعه‌دهندگان‌‌ سايت می‌باشد. هدف اين پژوهش بررسي ميزان دانش دانشجويان دانشگاه‌هاي علوم پزشكي غرب كشور در محيط‌هاي‌ وب 2 صورت گرفته‌‌‌‌است. روش‌ها: این پژوهش یک مطالعه توصيفي می‌باشد. جامعه آماري مطالعه، دانشجويان دانشگاه‌هاي علوم پزشكي غرب كشور بوده‌اند كه با استفاده از روش نمونه‌گيري تصادفي- طبقه‌اي، 303 نفر از آن‌ها به عنوان نمونه آماري انتخاب شده‌اند. ابزار جمع‌آوري داده‌ها، يك پرسش‌نامه محقق ساخته بوده كه روايي آن از نظر صاحب‌نظران حوزه علوم‌تربيتي و پايايي آن از ضريب‌ آلفاي‌ كرونباخ قابل‌ قبول (896). برآورد گرديده است. براي تجزيه و تحليل داده‌ها از آمار‌توصيفي و براي سنجش‌فرضيات از آزمون k² و ضرايب همبستگي فی و وي کرامر استفاده شده‌است. یافته‌ها: نتايج اين تحقيق بيانگر اين است كه ميزان دانش اكثر پاسخ‌گويان (58.4 درصد) در محيط‌هاي وب 2 كم و خيلي‌كم بوده و(7/26 درصد) از آن‌ها نيز در حد متوسط داراي دانش و مهارت بوده‌اند. همچنين بين ميزان دانش پاسخ‌گويان در زمينه محيط‌هاي وب 2 و معدل آن‌ها رابطه معنادار وجود داشته، در حالي كه بين مقطع تحصيلي پاسخ‌گويان و دانش‌‌‌ وب 20 رابطه معناداري مشاهده نشده است. نتیجه‌گیری: دانش دانشجويان در فناوري‌هاي وب 2 مطلوب نمي‌باشد. برنامه‌ريزي برای آشنايي با فناوري‌هاي‌نوين و ترغيب آن‌ها و استفاده از فناوري‌ها در فرايند يادگيري ضروري به‌نظر مي‌رسد.

Title: امکان‌سنجی استفاده از لجن خشک شده توسط تصفیه‌خانه‌هاي فاضلاب شهری با فرایند لجن فعال

http://hsr.mui.ac.ir/index.php/jhsr/article/view/74

مقدمه: یکی از گزینه‌هاي مطرح در خصوص دفع لجن، استفاده از آن در زمین‌هاي کشاورزي به عنوان کود می‌باشد ولي به دلیل احتمال وجود انواع میکروارگانیزم‌هاي بیماري‌زا، ممکن است باعث ایجاد طیف وسیعی از بیماري‌های میکروبی و انگلی در انسان و دام شود. هدف از انجام این تحقیق، بررسی امکان کاربرد لجن تولیدی در فرایندهای تصفیه فاضلاب شهری، برای حاصلخیزی زمین‌های کشاورزي با در نظر گرفتن استانداردهاي معتبر زیست محیطی و بهداشتی مي‌باشد. روش‌ها: در این مطالعه‌ی توصیفی- تحلیلی، سه تصفیه‌خانه فاضلاب واقع در شهرهای شهرکرد، فارسان و بروجن در استان چهارمحال و بختیاری به عنوان تصفیه‌خانه مدل، انتخاب شدند. دلیل انتخاب این تصفیه‌خانه مشابهت‌هایی بود که از نقطه‌نظر شرایط محلی، فرایندهای موجود در آن‌ها و نیز اهداف طرح وجود داشت. با توجه به کوهستانی بودن منطقه و دارا بودن آب و هوای سرد در زمستان و آفتاب داغ و سوزان در تابستان‌، دو فصل زمستان و تابستان به عنوان بازه زمانی طرح انتخاب گردیدند تا وضعیت لجن، در شرایط کمترین و بیشترین میزان تبخیر مورد بررسی قرار گیرد. در این تحقیق کلیفرم مدفوعی و کلیفرم کل به عنوان شاخص‌های میکروبی لجن تعیین شد و با استانداردهای سازمان حفاظت محیط زیست آمریکا از نظر بهداشتی مورد ارزیابی قرار گرفت.  یافته‌ها: نتایج حاصل از این تحقیق نشان مي‌دهد که مقادیر میانگین کلیفرم مدفوعی براي سه تصفیه‌خانه شهرکرد، فارسان و بروجن در فصل زمستان به ترتیب106 × 63/1، 106 × 93/1 و 106 × 98/1 و برای فصل تابستان به ترتیب 106 × 51/4، 106 × 75/5 و 106 × 23/9 مي‌باشد. نتیجه‌گیری: ارزیابی نتایج به دست آمده از این بررسی نشان می‌دهد که لجن بیولوژیکی تولید شده در هر سه تصفیه‌خانه در فصل زمستان پس از خشک کردن در شرایط طبیعی و بر مبنای استاندارد EPA در کلاس  Bواقع مي‌شوند. بنابراین بر اساس توصیه‌های این سازمان این گونه لجن‌ها را می‌توان با در نظر گرفتن محدودیت‌هایی که در استانداردها آمده است برای کشاورزی به کار گرفته شود. این در حالی است که كيفيت ميكروبي لجن‌های حاصل از این تصفیه‌خانه‌‌ها در فصل تابستان، پايين‌تر از استانداردهاي سازمان حفاظت محيط زيست آمريكا برای لجن کلاس B مي‌باشند و در نتيجه قابل استفاده در کشاورزی نمی‌باشند.

Title: بررسی تأثیر مشاوره زناشویی بر رضایت جنسی زوجین شهر شیراز

http://hsr.mui.ac.ir/index.php/jhsr/article/view/98

مقدمه: امروزه علت شکست 50 درصد از ازدواج‌ها، عدم رضایت جنسی می‌باشد. این مطالعه با هدف تأثیر مشاوره زناشویی بر رضایت جنسی زوجین مراجعه‌ کننده به مرکز بهداشتی درمانی نادر کاظمی شهر شیراز انجام شد. روش‌ها: این مطالعه نیمه تجربی با روش نمونه‌گیری آسان روی (60 زوج) کلیه زوجین تازه ازدواج کرده شهر شیراز که 6 ماه قبل جهت دریافت گواهی ازدواج دایم به مرکز بهداشتی درمانی نادر کاظمی شهر شیراز مراجعه نمودند. انجام شد. زوجین با تقسیم تصادفی در دو گروه آزمون و شاهد قرار گرفتند. داده‌ها با پرسش‌نامه‌های مشتمل بر دو قسمت، مشخصات دموگرافیک (دارای 6 سؤال) و پرسش‌نامه استاندارد رضایت جنسی ISS استفاده شد. در مرحله پيش آزمون داده‌ها با پرسش‌نامه كه روايي و پايايي آن مورد بررسي و تأييد قرار گرفته بود در دو گروه گردآوري گرديد. سپس برنامه مداخله آموزشي 4 جلسه‌اي براي گروه آزمون انجام شد. پس آزمون یک ماه بعد از دو گروه به عمل آمد و داده‌ها با آزمون های آماری تجزيه و تحليل شد. یافته‌ها: این مطالعه نشان داد که 67/46 درصد (28 نفر) زنان گروه آزمون. 33/53 درصد (32نفر) زنان گروه شاهد در رده سنی 24- 20 سال و نیز 3/53 درصد مردان (32نفر) گروه آزمون و 50 درصد مردان (30 نفر) گروه شاهد در رده سنی 29- 25 سال بودند بودند و بین دو گروه در رده‌های سنی ذکر شده تفاوت آماری معنی‌دار مشاهده نشد (05/0<P). تفاوت معنی‌داری در رضایت جنسی در زنان و مردان بین دوگروه آزمون و شاهد در پس آزمون دیده شد (002/0=P). نتیجه‌گیری: مشاوره زناشویی بر کیفیت روابط جنسی تأثیر گذارده و رضایت جنسی زوجین را افزایش می‌دهد. مشاوره زناشویی با پيشگيري از وقوع اختلالات جنسي و بيماري‌هاي جنسي یکی از کارآمدترین روش‌های آموزش بهداشت و سلامتی برای کمک به افراد و زوجین است

Title: وضعیت رفتار پیاده‌روی در بیماران مبتلا به دیابت تیپ 2 شهر یزد بر اساس مدل اعتقاد بهداشتی

http://hsr.mui.ac.ir/index.php/jhsr/article/view/136

مقدمه: امروزه نقش مثبت فعاليت‌هاي جسماني و ورزشي به خصوص پیاده‌روی در سلامت جامعه بشري و در پيش‌گيري و كنترل بسياري از بيماري‌ها از جمله بيماري ديابت ثابت شده است.هدف از این بررسی به منظور تعیین پیش‌بینی‌کننده‌های رفتار پیاده روی در بیماران مبتلا به دیابت تیپ 2 شهر یزد بر اساس مدل اعتقاد بهداشتی صورت گرفت. روش‌ها: در این مطالعه‌ا‌ی توصیفی ـ مقطعی، تعداد 111 نفر ازبیماران دیابتی مراجعه کننده به مرکز تحقیقات دیابت شهر یزد بصورت تصادفی انتخاب شدند.ابزار جمع‌آوری داده‌ها پرسش‌نامه‌ای مشتمل بر سازه‌های مدل اعتقادبهداشتی به انضمام متغیرهای دموگرافیک و سؤالات انجام رفتار پیاده‌روی بود. داده‌ها با استفاده از نرم‌افزار SPSS و به کار‌گیری آمار توصیفی، آزمون‌های ضریب همبستگی و رگرسیون مورد تجزیه و تحلیل قرار گرفت. یافته‌ها: افراد مبتلا به دیابت نوع 2 (78 زن و 33 مرد) با میانگین سنی 44/7±18/49 در این مطالعه شرکت کردند. 102 نفر متأهل و 55 نفر از آنها تحصیلات زیر دیپلم داشتند. میانگین نمره آگاهی و رفتار پیاده‌روی به ترتیب 22/3± 93/6 و 73/3±46/4 به دست آمد. افراد مورد مطالعه تنها 12/43 درصد از ماکزیمم نمره قابل اکتساب آگاهی و85/31 درصد از ماکزیمم نمره رفتار پیاده‌روی را کسب کردند. بر اساس نتایج این مطالعه بین رفتار پیاده‌روی و آگاهی (02/0> P و 221/0=r)، رفتار و شدت درک‌شده (03/0> P و 201/0=r)، رفتار و منافع درک شده (01/0> P و 234/0=r ) همبستگی معنادار وجود دارد. نتیجه‌گیری: مدل اعتقاد‌بهداشتی می‌تواند علل ضعف انجام پیاده‌روی را پیش‌گویی کند و به عنوان چارچوبی جهت اجرای مداخلات آموزشی به منظور کنترل بیماری دیابت مورد استفاده قرار گیرد. واژه‌های‌ کلیدی: مدل اعتقاد بهداشتی، پیاده‌روی، دیابت نوع 2

Title: مقایسه روش تعیین مقدار اکسیژن مورد نیاز شیمیایی (COD) در فاضلاب‌های شور و غیر شور

http://hsr.mui.ac.ir/index.php/jhsr/article/view/51

مقدمه: در صورت حضور یون‌هایی مانند کلراید، برماید و یداید، در نمونه‌های فاضلاب و به ویژه فاضلاب‌های شور، مقدار گزارش شده به عنوان مقدار اکسیژن مورد نیاز شیمیایی (COD یا Chemical oxygen demand)، مقدار حقیقی نمی‌باشد. هدف از این مطالعه تعیین میزان خطای آزمایش اندازه‌گیری COD ارایه شده در "روش‌های استاندارد آزمایشات آب و فاضلاب" در حضور یون کلراید برای فاضلاب‌های شور و غیر شور با غلظت‌های مختلف COD بود. روش‌ها: آزمایش به دو شکل انجام شد. روش اول بدون هر گونه حذف یون کلراید بود و روش دوم از روش حذف یون کلراید پیشنهادی در "روش‌های استاندارد آزمایشات آب و فاضلاب" استفاده شد. در این روش برای حذف اثر تداخل یون کلراید از نسبت HgSO4:Cl، 10:1 استفاده شد. تعداد نمونه‌ها 22 عدد بود و تمامی آن‌ها با دو بار تکرار سنجش گردید. گستره غلظت NaCl و COD در نمونه‌ها به ترتیب در محدوده g/L 5-5/1 و mg/L 1500-400 متغیر بود. نمونه‌ها به دو بخش فاقد نمک به عنوان شاهد و حاوی نمک تقسیم شد. یافته‌ها: مقدار خطا در روش اول (بدون حذف شوری) معادل 16 درصد و در روش دوم (پس از حذف شوری) معادل 2/10 درصد بود. در روش دوم 6/63 درصد از داده‌های قرائت شده از مقدار واقعی کمتر بود؛ در حالی که در روش اول 4/36 درصد از داده‌ها از مقدار واقعی کمتر بودند. نتیجه‌گیری: حضور یون کلراید در فاضلاب‌های شور سبب ایجاد خطا در مقدار قرائت COD شد که این خطا گاه مثبت و گاه منفی است. در غلظت‌های بالای COD، مقدار خطا بیشتر است که دلیل آن را می‌توان از یک طرف به اکسیداسیون یون کلراید توسط دی کرومات و از طرفی به مقدار مواد آلی باقی‌مانده و اکسید نشده مرتبط دانست.

Title: بررسی تأثیر آموزش هدف‌دار بر تغییر آگاهی و نگرش مادران باردار در زمینه مسمومیت حاملگی

http://hsr.mui.ac.ir/index.php/jhsr/article/view/64

مقدمه: مسمومیت حاملگی با 14 درصد مرگ و میر مادران در كشورهای جهان سوم همراه است. از آن جا كه شناسایی جمعیت مادران در معرض خطر و اقدامات مناسب با پیش آگاهی مناسب مادر و جنین همراه می‌باشد، داشتن آگاهی كافی بهداشت كاران خانواده و مادران می‌تواند به كنترل بهتر این بیماری و عوارض آن بیانجامد. در این بررسی سطح آگاهی و نگرش مادران قبل و بعد از دوره‌های آموزشی هدف‌دار مورد بررسی قرار گرفت. روش‌ها: در یك كارآزمایی قبل و بعد، آگاهی و نگرش 70 زن باردار با سواد خواندن و نوشتن، قبل و بعد از برگزاری جلسه آموزشی هدف‌دار در مورد فشار خون حاملگی مورد بررسی قرار گرفت. در این بررسی از پرسش‌نامه خود ایفای مشتمل بر 15 سؤال آگاهی‌سنج و 6 سؤال نگرش‌سنج استفاده شد. داده‌ها در نرم‌افزار SPSS مورد تجزیه و تحلیل قرار گرفت. یافته‌ها: در این بررسی نمره آگاهی مادران قبل و بعد از مطالعه به ترتیب 3/20 ± 44 و 5/16 ± 4/76 بود، میانگین و انحراف معیار نمره نگرش مادران نیز از 7/19 ± 5/57 به 4/8 ± 3/92 در پایان مطالعه افزایش یافت. در این مطالعه نمره آگاهی نگرش مادران با تعداد زایمان، تعداد حاملگی و سن مادران همبستگی معنی‌داری نداشت. همچنین 4/52 درصد از مادران آگاهی ضعیف و بسیار ضعیف داشتند. نتیجه‌گیری: آگاهی و نگرش زنان باردار به دنبال آموزش‌های هدف‌دار افزایش معنی‌داری یافت. مقایسه یافته‌های این مطالعه با آگاهی ضعیف بیش از 50 درصد از زنان باردار در ابتدای مطالعه، لزوم تدوین برنامه آموزشی برای جمعیت‌های خاص در معرض خطر بالا و بهداشت‌ كاران خانواده را ضروری می‌سازد.

Title: بررسی دریافت ویتامین‌های آنتی‌اکسیدانی در زنان ورزشکار در دو منطقه اجتماعی‌-‌اقتصادی مختلف

http://hsr.mui.ac.ir/index.php/jhsr/article/view/123

مقدمه: ورزش تولید رادیکال‌های آزاد و پراکسیدان‌های لیپیدی را افزایش می‌دهد که باعث آسیب اکسیداتیو و در نتیجه آسیب بافتی می‌شود. ویتامین C و E ممکن است پراکسیدان‌های لپیدی افزایش یافته در ورزش را کاهش دهند.  مطالعه حاضر به بررسی وضعیت دریافت ویتامین‌های آنتی‌اکسیدانی در زنان ورزشکار مراجعه‌کننده به سالن‌های ورزشی آئروبیک در دو منطقه اجتماعی‌-‌اقتصادی مختلف شهر اصفهان می‌پردازد. روش‌ها: در اين مطالعه توصيفي 99 زن در رنج سني 50-15 سال با نمونه‌گيري تصادفي انتخاب شدند. با تكميل پرسشنامه يادآمد 24 ساعته براي 2 روز متوالي اطلاعات غذايي افراد مورد مطالعه به دست آمد. در نهايت ميانگين دريافت ویتامین C و ویتامین E اندازه‌گيري و با مقدار RDA و همچنین بین دو گروه مقايسه شد. جهت آناليز داده‌ها از نرم‌افزار  N4وSPSS  استفاده شد. یافته‌ها: میانگین دریافت ویتامین E در هر دو منطقه‌ی اقتصادی بالا (001/0 P=) و منطقه‌ی اقتصادی پایین P=<0/001)) کمتر از مقدار توصیه شده است و میانگین دریافت ویتامین E در افراد منطقه اقتصادی بالا به طور معنی‌داری از افراد منطقه اقتصادی پایین بیشتر است. (033/0P=) میانگین دریافت ویتامین Cدر هر دو منطقه‌ی‌اقتصادی بالا (004/0P=) و منطقه‌ی اقتصادی پایین (006/0P=) بیشتر از مقدار توصیه شده می‌باشد. اما اختلاف معنی‌داری بین میانگین دریافت ویتامین Cدر این دو منطقه مشاهده نشد (098/0P=). نتيجه‌گيري: دريافت ویتامین E در زنان ورزشكار پايين است ارائه راهكارهاي لازم براي بهبود وضعيت دريافت ویتامین E ضروري به ‌نظر مي‌رسد.

Title: مقایسه کمی و کیفی مقدار مواد خارجی موجود در کمپوست کارخانه کمپوست خمین و تهران در سال 1387

http://hsr.mui.ac.ir/index.php/jhsr/article/view/104

مقدمه: کیفیت کمپوست تولیدی، یکی از پارامترهای مهم و تأثیرگذار در کیفیت و فروش کمپوست بوده، مواد خارجی موجود در کمپوست اهمیت ویژه‌ای دارند. در این مطالعه، کیفیت مواد خارجی موجود در کمپوست تولیدی کارخانه‌های خمین و تهران مطالعه شد. روش‌ها: این مطالعه به مدت 9 ماه بر روی کمپوست تولیدی در کارخانه‌های کمپوست خمین و تهران، با نمونه‌برداری مرکب تعداد 10 نمونه از کمپوست تولیدی برداشته شد و مواد خارجی آن از قبیل شیشه، پلاستیک، فلز، مواد خارجی و سنگریزه مورد آنالیز قرار گرفت و از آزمون t برای مقایسه میانگین داده‌ها استفاده شد. یافته‌ها: میانگین درصد شیشه، پلاستیک، فلز، مواد خارجی و سنگریزه در کمپوست تولیدی تهران 62/0، 22/0، 0، 84/0 و 24/2 درصد به دست آمد. در کمپوست خمین به ترتیب 17/1، 17/0، 02/0، 36/1 و 61/3 بود، که در محدوده استانداردهای مورد نظر می‌باشد. همچنین با توجه به آزمون آماری اختلاف معنی‌داری بین میانگین پلاستیک نمونه‌های کمپوست خمین و تهران مشاهده نشد، ولی میانگین سایر اجزاء اختلاف معنی‌دار با هم داشت (0001/0 < P). نتیجه‌گیری: به طور کلی، كمپوست تولیدی کارخانه‌های تهران و خمین، مشكل خاصی از لحاظ مواد خارجی نداشت و در محدوده اکثر استانداردها قرار داشت. با این حال، چون مقدار مواد خارجی کمپوست حتی در مقادیر کم نیز موجب عدم ترغیب کشاورزان به خرید کمپوست می‌شود، با کاهش مواد خارجی توسط برنامه‌های تفکیک از مبدأ و ممانعت پسماند مخلوط به کارخانه کمپوست، باعث بهبود کیفیت و ورود آن به بازار مصرف خواهد بود.

Title: ارتباط میزان مصرف میوه‌جات و سبزیجات و فعالیت بدنی با سطح هموگلوبین گلیکوزیله در افراد مبتلا به دیابت نوع 2

http://hsr.mui.ac.ir/index.php/jhsr/article/view/126

مقدمه: امروزه دیابت یکی از شایع‌ترین بیماری های متابولیک و عامل مهمی برای مرگ و میر و ناتوانی در جوامع مختلف از جمله ایران می‌باشد. دیابت درمان قطعی ندارد ولی می‌توان با تأمین رژیم غذایی مناسب از پیشرفت و بروز عوارض آن جلوگیری کرد. هدف این مطالعه بررسی ارتباط میزان مصرف میوه‌جات و سبزیجات با سطح هموگلوبین گلیکوزیله (HbA1C) در بیماران مبتلا به دیابت نوع 2 می‌باشد. روش‌ها: این مطالعه به صورت مقطعی بر روی 105 بیمار مبتلا به دیابت در سال 1389 انجام گردید. نمونه‌ها به روش نمونه‌گیری آسان انتخاب شدند. داده‌های عمومی و دریافت غذایی با استفاده ازپرسشنامه‌های عمومی و بسامد خوراک (FFQ) و میزان هموگلوبین گلیکوزیله بیماران با انجام آزمایش خون تعیین گردید. پردازش اطلاعات با استفاده از نرم‌افزارهای4  Nutritionist  و SPSS انجام شد. یافته‌ها: با افزایش 10 گرم مصرف سبزیجات به ‌تنهایی و یا افزایش 10 گرم مصرف میوه‌جات وسبزیجات به‌ طور هم‌ زمان به ترتیب میزان هموگلوبین گلیکوزیله 0.03درصد و 0.02درصد به طور معنی‌داری   p=0.049)و (p=0.014کاهش می‌یابد. نتيجه‌گيري: افرادی ديابتي که از میوه‌جات و سبزیجات بیشتری استفاده مي‌كنند سطح هموگلوبین گلیکوزیله پایین‌تری دارند.

Title: بررسی آلودگی صوتی در یک مجتمع تحقیقاتی جنب فرودگاه مهرآباد

http://hsr.mui.ac.ir/index.php/jhsr/article/view/89

مقدمه: صدا يکي از عوامل زيان آور محيط‌هاي صنعتي است که اختلالاتي از قبيل نقص شنوايي‌، پر فشار خونی‌، آزردگی‌، اختلالات خواب‌، افزایش تعداد تنفس و مصرف اکسیژن‌ را به وجود مي‌آورد. هدف این پژوهش اندازه‌گيري و ارزیابی میزان ‌مواجهه ‌پرسنل با صدا در واحدهاي مختلف صنعتي و اداري مجتمع دانشگا‌هي هوا و فضا در جنب فرودگاه مهرآباد بود. روش‌ها: در این مطالعه به دليل تنوع هواپيماهاي موجود در سيستم حمل و نقل هوايي و متغير بودن تعداد پروازها در طول روز دوزيمتري فردي هشت ساعته، در يک دورة يک هفته‌اي و همچنين آناليز اکتا و باند در سالن تونل باد انجام شد. صداسنج (مدل 440CEL) و دوزيمتر282(مدل-CEL ) جهت اندازه‌گيري استفاده شد. داده‌ها با‌ استاندارد کميته فني بهداشت حرفه‌اي ایران و انجمن دولتي متخصصين بهداشت صنعتي آمريكا (ACGIH -2005) مقايسه گرديد. یافته‌ها: مقادیر به دست آمده از دوزيمتري سر و صدا در سالن‌هاي ساختdB  7/96، آهنگري و جوشکاري dB 6/99، آيروديناميک d‌B94، مدل سازي و نجاري dB 6/99، ورق کاري dB 95، کارگاه تونل باد dB 94، ساختمان‌هاي اداري ستاد dB 77، دفتر طراحي dB 77و ايستگاه نگهباني dB 76 بود. نتايج آناليز اکتاوباند سالن تونل باد در فرکانس‌هاي 125، 250، 500، 1000، 2000، 4000 و 8000 هرتز به ترتيب 1/97، 5/95، 1/93، 2/90، 5/85، 6/77 و 46/68 دسي بل بدست آمد. نتیجه‌گیری: میزان سرو صدا بالاتر از میزان‌های استاندارد ACGIH و کمیته فنی بهداشت حرفه‌ای کشور می‌باشد و برای پیشگیری از پیامد‌های مواجهه با سروصدا‌ توصیه‌هایی ارایه گردید. واژه‌های ‌کلیدی: آلودگي صوتي، صداسنجي، دوزیمتری صدا، فرودگاه مهر آباد

Title: مقایسه اثربخشی برنامه آموزش تغذیه بر اساس مدل اعتقاد بهداشتی با آموزش سنتی در افزایش وزن دوران بارداری

http://hsr.mui.ac.ir/index.php/jhsr/article/view/99

مقدمه: يكي از تغييرات مهم دوران بارداری افزايش وزن زنان باردار به عنوان يكي از شاخص‌هاي معتبر تغذيه است كه بر اساس شاخص توده بدني (BMI) سنجيده مي‌شود. اين مطالعه با هدف مقایسه تأثير برنامه آموزش تغذيه بر اساس مدل اعتقاد بهداشتی با آموزش کلاسیک بر افزايش وزن توصيه شده دوران بارداري در زنان باردار در شهر گناباد انجام شد. روش‌ها: مطالعه نيمه تجربي روی 110 زن باردار مراجعه‌كننده به مراكز بهداشتي درماني شهر گناباد در سال 1388 در دو گروه آزمون (54 نفر) و كنترل (56 نفر) انجام شد . انتخاب گروه آزمون و شاهد به این صورت بود که اولین نفر به تصادف در گروه آزمون و نفر بعدی در گروه شاهد قرار می‌گرفت و این روند تا پایان نمونه‌گیری ادامه یافت در مرحله پيش آزمون داده‌ها توسط پرسش‌نامه محقق ساخته كه روايي و پايايي آن مورد بررسي و تأييد قرار گرفته بود در دو گروه در نوبت اول مراقبت دوران بارداري جمع‌آوري گرديد. سپس برنامه مداخله آموزشي 2 جلسه‌اي متناسب با سازه‌های مدل اعتقاد بهداشتی براي گروه آزمون و 2 جلسه آموزشی بر اساس آموزش‌های سنتی انجام شد. پس آزمون در آخرين نوبت مراقبت بارداري از دو گروه به عمل آمد و داده‌ها توسط آزمون‌هاي آماري تجزيه و تحليل شد. یافته‌ها: آزمون‌های آماري تفاوت معنی داری را بين تحصيلات، تعداد بارداري، سابقه سقط، شغل و ميانگين سني در دو گروه نشان نداد. بعد از مداخله مهم‌ترين راهنماي عمل خارجي گروه آزمون، همسر و گروه كنترل، كاركنان بهداشتي درماني بود. آزمون‌های آماري تفاوت معني‌داري را بين دو گروه در خصوص كسب وزن مطلوب در اين دوران را نشان مي‌داد. نتيجه‌گيري: کاربرد الگوی اعتقاد بهداشتی در جهت آموزش تغذیه به منظور افزایش وزن توصیه شده در زنان باردار نتایج موفقی را در مقایسه با آموزش‌های سنتی دارد.

Title: بررسی میزان حذف ترکیبات عامل سمیت نفت خام در آب آشامیدنی توسط فرایند ازن زنی

http://hsr.mui.ac.ir/index.php/jhsr/article/view/68

مقدمه: نفت خام ترکیبی بسیار پیچیده است و حاوی تعداد زیاد هیدروکربن با اثرات مضری مثل سرطان‌زایی و اثرات خونی می‌باشد. در این مطالعه فرایند ازن زنی جهت کاهش سمیت نفت خام مورد استفاده قرار گرفت. روش‌ها: نمونه استاندارد قسمت محلول نفت خام با اختلاط یک قسمت نفت خام با 9 قسمت آب تهیه گردید. جهت آزمون سمیت از غلظت‌های مختلف، قسمت محلول نفت خام و لارو ماهی قزل‌آلا استفاده شد. قبل و بعد از فرایند ازن زنی با غلضت‌های مختلف، آزمون سمیت و کربن آلی کل، روی نمونه‌ها انجام گردید. یافته‌ها: با افزایش زمان آزمون سمیت، اختلاف بیشتری در نتایج حاصل از آزمون سمیت مشاهده شد. میزان غلظت کشنده 50 درصد 96 ساعته و کل کربن آلی قبل از فرایند ازن زنی برابر با 60 و 98/54 میلی‌گرم بر لیتر بود. بعد از فرایند ازن زنی با مقادیر 1 و 10 میلی‌گرم بر لیتر، میزان غلظت کشنده 50 درصد 96 ساعته به 3/113 و 5/205 و کل کربن آلی به 8/42 و 04/40 میلی‌گرم بر لیتر رسید. نتیجه‌گیری: غلظت‌های بالاتر ازن جهت كاهش سمیت، تأثیر زیادی داشت، ولی در كاهش میزان محتوی كربن آلی كل تأثیر قابل توجهی مشاهده نشد. همچنین برای بررسی تأثیر یک فرایند تصفیه مؤثر برای حذف آلاینده‌های خاص، استفاده از آزمایش‌های زیست آزمونی می‌تواند نتایج ارزشمندی داشته باشد و به عنوان مکمل پیشنهاد می‌گردد.

Title: بررسي ارتباط آگاهي و نگرش روستاييان با منابع کسب اطلاعات در زمينه برنامه پزشك خانواده در شهرستان شهركرد سال 1388

http://hsr.mui.ac.ir/index.php/jhsr/article/view/87

مقدمه: برنامه پزشک خانواده و نظام ارجاع يکي از برنامه­هاي مهم در بخش بهداشت و درمان کشور است که از سال 84 به اجرا درآمده است. بهداشت و سلامت پديده­اي خودجوش است و بايد اطلاعات مربوطه در اين زمينه به افراد داده شود. هدف از این مطالعه­ بررسي ارتباط بين آگاهي و نگرش روستاييان با منابع کسب اطلاعات در مورد برنامه پزشک خانواده و بيمه روستايي در مناطق روستايي شهرستان شهرکرد در سال 1388 بود. روش‌ها: اين پژوهش از نوع توصيفی -  تحليلي بود که تعداد 1100 نفر (5/45 % مرد و 5/54% زن( از افراد تحت پوشش برنامه پزشک خانواده درمناطق روستايي شهرستان شهرکرد به روش نمونه­گيري به صورت چند مرحله­اي انتخاب شدند. جهت جمع آوري اطلاعات، پرسش­نامه استفاده شد.  در نهایت داده­ها بوسیله آزمون­هاي آماري t-test  و  ANOVA  آنالیز شد. يافته‌ها: نتايج حاصل از پژوهش نشان داد که ميانگين سني افراد مورد بررسي 23/34 سال بود، تنها به 51 % از روستاييان، موضوعات مربوط به برنامه پزشک خانواده آموزش داده شده بود و بين ميانگين نمره آگاهي و نگرش روستاييان با آموزش­هاي داده شده ارتباط معني­داری مشاهده شد(0.001 ,P<0.05P<). بيشترين درصد روستاييان اطلاعات خود را از (34 درصد) بهورزان و کارمندان مراکز بهداشتي درماني دريافت نموده بودند، همچنين بين منابع کسب اطلاعات و آگاهي و نگرش روستاييان تفاوت معني­داري مشاهده شد(0.001(P<. نتيجه‌گيري: ميزان اطلاع رساني به افراد در مورد برنامه در حد متوسط مي­باشد، با توجه به اينکه روستاييان بيشترين اطلاعات خود را از بهورزان دريافت کرده بودند، بنابراين اجراي برنامه­هاي آموزشي براي پزشکان و  بهورزان و انتقال به ساير افراد جامعه مي­تواند موجب ارتقاي کيفيت و اثربخشي اين برنامه گردد.

Title: بررسی فشار خون و ارتباط آن با نمایه توده بدن در ساکنین روستاهای شهرستان اصفهان

http://hsr.mui.ac.ir/index.php/jhsr/article/view/145

مقدمه: چاقی یک فاکتور خطر مستقل برای ابتلا به بیماری‌‌های قلبی- عروقی، دیابت نوع 2، بیماری‌های کیسه صفرا، سرطان‌های مختلف، التهاب مفاصل و مرگ و میر می‌باشد. افزایش وزن طی بزرگسالی به ویژه در دهه‌های سوم و چهارم زندگی با افزایش فشار خون همراه می‌باشد. روش‌ها: در اين مطالعه مقطعي تعداد 1493 (1159 زن و 334 مرد) فرد 16 تا 85 ساله در 12 منطقه روستایی اطراف اصفهان به طور تصادفي از بين افراد قابل دسترسي مورد بررسی قرار گرفتند و متغیرهای فشار خون، سن، قد، وزن، جنس و ارتباط بين آن‌ها مورد مطالعه قرار گرفت. BMI از رابطه وزن و قد محاسبه شد. آناليز آماري اطلاعات به وسیله نرم‌افزار SPSS11.5 و با استفاده از آزمون ANOVA و محاسبه ضريب همبستگي پيرسون براي بررسي ارتباط‌‌ها صورت گرفت. یافته‌ها: میانگین نمایه توده بدن در این مطالعه 37/23 کیلوگرم بر متر مربع، فشار سیستولی 122 و فشار دیاستولی 76 میلی‌متر جیوه بود. در اين مطالعه بيش از نصف افراد شرکت کننده BMI در محدوده طبيعي 24-20 و درصد کمی BMI بالاتر از 40 را دارا بودند. فشار خون سيستوليک و دياستوليک ارتباط معني‌داري با BMI داشت (0001/0 > P، 202/0 = r). بر اساس آزمون ANOVA فشار خون سيستوليک و دياستوليک به طور معني‌داري در بين گروه‌هاي مختلف BMI متفاوت بود (0001/0 > P). نتیجه‌گیری: نتايج اين تحقيق نیز ارتباط فشار خون و BMI را تأیید می‌کند. به نظر می‌رسد افزایش وزن با احتباس نمک و مایعات، باعث افزایش فشار خون می‌شود، بنابراین کنترل وزن جزء مهمی در کنترل فشار خون محسوب می‌شود. کنترل فشار خون از طریق پیشگیری اولیه با شناسایی موارد جدید و در معرض خطر امکان پذیر است.

Title: بررسی دریافت ریز مغذی‌ها و درشت مغذی‌ها در رژیم غذایی دانشجویان دختر دانشگاه علوم پزشکی اصفهان و مقایسه آن با استاندارد‌های تعیین‌شده

http://hsr.mui.ac.ir/index.php/jhsr/article/view/155

مقدمه: تغذیه مناسب به ویژه دریافت انرژی و مواد مغذی ضروری و کافی در دختران جوان به خصوص دانشجویان بسیار حایز اهمیت است. مطالعه حاضر با هدف تعیین دریافت ریز مغذی‌ها و درشت مغذی‌ها و مقایسه آن با استاندارد‌های تعیین‌شده، بر روی دانشجویان دختر دانشگاه علوم پزشکی اصفهان انجام شد. روش‌ها: این مطالعه مقطعی روی 100 نفر دختر دانشجوی 25-18 ساله در سال تحصیلی 88-1387 انجام شد. داده‌های تن‌سنجی به روش استاندارد، اندازه‌گیری شد و داده‌های غذایی با استفاده از 2 یادآمد 24 ساعت خوراک جمع‌آوری شد و توسط 2food prossesor  مورد تجزیه و تحلیل قرار گرفت. یافته‌های مربوط به دریافت مواد مغذی با مرجع میزان توصیه شده مواد مغذی (2008DRI, Dietary reference intakes )، مورد مقایسه قرار گرفتند. یافته‌ها: 1/61 درصد افراد مورد مطالعه خوابگاهی و 7/12 درصد متأهل بودند. شیوع چاقی و اضافه وزن در افراد مورد بررسی 9/6 درصد و چاقی شکمی 1/46 درصد بود. میانگین فشار خون سیستولی 6/15 ± 2/105 میلی‌متر جیوه و دیاستولی 4/10 ± 2/69 میلی‌متر جیوه بود. در مجموع 9/3 درصد مبتلا به پرفشاری خون بودند. از بین مواد مغذی دریافتی، میزان دریافت 12B، ریبوفلاوین، فولات، منیزیم، پتاسیم و کلسیم در مقایسه با مقادیر توصیه شده DRI ناکافی و میزان دریافت E، C، پانتوتنیک اسید، تیامین، نیاسین، فسفر و روی بیش از مقادیر توصیه شده بود و وضعیت دریافت درشت مغذی‌ها، انرژی دریافتی، A، پیرودوکسین، آهن و سلنیوم مطابق با مقادیر توصیه شده بود. نتیجه‌گیری: از بررسی نتایج مطالعه حاضر مشخص گردید که دریافت درشت مغذی‌ها در دانشجویان مشکل چندانی ندارد و عدم تعادل، عمدتاً در گروه ریزمغذی‌ها است. بنابراین مصرف گروه‌های مهم غذایی مانند لبنیات، سبزی و میوه که منابع عمده ریز مغذی‌ها می‌باشند در این جامعه باید افزایش یابد. با توجه به اهمیت تغذیه مناسب در این رده سنی و اثرات آن بر سلامت آینده جامعه، به نظر می‌رسد باید توجه بیشتری به آموزش و فراهم آوردن امکانات برای عملکرد مناسب تغذیه‌ای دانشجویان مبذول شود.

Title: بررسی سبک زندگی معلمان شاغل درشهرستان گرگان در سال 1388

http://hsr.mui.ac.ir/index.php/jhsr/article/view/134

مقدمه: سبك زندگي، فعاليت عادي و معمول روزانه است كه افراد آن‌ها را در زندگي خود به طور قابل قبول پذيرفته‌اند که بر روي سلامتي افراد تأثير مي‌گذارند. اين مطالعه با هدف بررسي سبك زندگي معلمان شهرستان گرگان در سال 1388 انجام گرفت. روش‌ها: پژوهش حاضر  توصيفي تحليلي، 336 معلم به روش نمونه‌گیری طبقه‌ای‌ـ خوشه‌ای در مقاطع ابتدايي راهنمايي، دبيرستان و پيش دانشگاهي انتخاب شدند. اطلاعات با پرسش‌نامه جمع‌آوری گردید و با نرم‌افزار آماري 5/11SPSS  با استفاده ازروش‌های آماری توصیفی و آزمون من ويتني، t مستقل، كروسكال واليس و ضريب همبستگي پيرسون مورد تجزيه و تحليل قرار گرفت. ضریب اطمینان 95 درصد درنظرگرفته شد. یافته‌ها: در این پژوهش 268 نفر (84 درصد) داراي سبك زندگي نيمه مطلوب بودند. 251نفر 7)/74 درصد ) از كمربند ايمنی در هنگام رانندگي استفاده مي‌كردند. 14 نفر (2/4 درصد ) افراد سيگاري 80 نفر (6/23 درصد ) به طور مرتب ورزش مي‌كردند. 181 نفر )54 درصد( داراي اضافه وزن يا چاقي بر اساس معيار نمايه توده بدني بودند. 148 نفر )1/44 درصد( افراد در زمينه تغذيه سالم و 239 نفر )1/71 درصد( نيز در زمينه تغذيه غيرسالم داراي وضعيت مطلوب بودند. 302 نفر (9/82 درصد( در زمينه كنترل استرس در وضعيت نيمه مطلوب بودند. بين سبك زندگي و متغير مقطع تدریس (p=0.05) و بين انجام ورزش مرتب و جنسيت رابطه معني‌دار مشاهده شد (p=0.002). نتیجه‌گیری: با توجه به اينكه اكثريت افراد مورد بررسي داراي سبك نيمه مطلوب و غير مطلوب بودند مداخله سازمان يافته و گسترده و منطبق با شرایط افراد مورد بررسی ضروری به نظر می‌رسد.

Title: تعیین سن منارک و ارتباط آن با قد فعلی و وضعیت اجتماعی –اقتصادی در دختران شهر آق قلا در سال 1388

http://hsr.mui.ac.ir/index.php/jhsr/article/view/135

مقدمه: سنی که در آن اولین قاعدگی رخ می‌دهد به عنوان سن منارک در نظر گرفته می‌شود و شاخص خوب رسیدگی زن محسوب می‌شود.این پژوهش‌ با هدف تعیین سن منارک و ارتباط آن با قد فعلی و وضعیت اجتماعی– اقتصادی در دختران دانش‌آموز شهر آق قلا انجام گرفت. روش‌ها: در این پژوهش توصیفی تحلیلی 145 نفر دانش‌آموز مقطع راهنمایی شهر آق قلا در سال 1388از طریق سرشماری وارد مطالعه شدند. ابزار گردآوري اطلاعات پرسش‌نامه‌اي روا وپايا به‌ همراه متر پلاستيكي و ترازوي استاندارد بود. داده‌ها با استفاده از آزمون‌های‌ t مستقل، ANOVA‌، و ضریب همبستگی پیرسون نرم افزار آماری SPSS11/5 تجزیه و تحلیل گردید.  يافته‌ها: میانگین و انحراف معیار سن شروع منارك 98/0±15/12 سال بود. میانگین و انحراف معیار قد و وزن فعلی آنان برابر به ترتیب برابر با 37/5±35/156 سانتی متر و 34/9±51/49 کیلو‌گرم بود. همچنین میانگین و انحراف معیار نمایه توده بدنی آنانBody Mass Index  (BMI) 4/3±2/20 کیلوگرم بر متر مربع بود. ارتباطي بين سن منارك نمونه‌ها با قد فعلي، وزن فعلي، نمايه توده فعلي، سطح تحصيلات والدين، رتبه تولد، بعد خانوار، محل سكونت و قوميت جد نمونه‌ها مشاهده نشد ( p>0.05از لحاظ آماری تفاوت معنی‌داری بين شروع منارك و فصول سال مشاهده شد(P<0.05). نتیجه‌گیری‌: جهت استنتاج و دسترسي به نتايج قطعي‌تر مطالعه گسترده‌تربه روش آينده‌نگر و با حجم‌ نمونه بیشتر در سطح شهرستان با در نظر گرفتن تمامي عوامل مرتبط جهت برنامه‌ریزی بهداشت دوران بلوغ ضروری به نظر می‌رسد. واژه‌های‌ كليدي: سن منارك، بلوغ،دانش‌آموز، آق قلا.

Title: تأثیر تمرین‌هاي اصلاحی بر اختلال‌هاي عضلانی- اسکلتی

http://hsr.mui.ac.ir/index.php/jhsr/article/view/176

مقدمه: اختلال‌هاي عضلانی– اسکلتی مرتبط با کار عمده‌ترین عامل از دست رفتن زمان کار، افزایش هزینه‌ها و آسیب‌های انسانی نیروی کار به شمار می‌رود. هدف از تحقیق حاضر، بررسی شیوع اختلال‌هاي عضلانی - اسکلتی مرتبط با کار پرستاران بیمارستان الزهرا و تأثیر 8 هفته تمرین‌هاي اصلاحی منتخب و مداخله‌هاي ارگونومی بر آن‌ها بود. روش‌ها: از پرسش‌نامه نوردیک برای مطالعه شیوع اختلال‌هاي عضلانی- اسکلتی مرتبط با کاراستفاده شد و همچنین برای ارزیابی سطوح مواجهه با خطر اختلال‌هاي عضلانی - اسکلتی در 6 وظیفه کاری پرستاران عکس‌برداری و از روش ارزیابی سریع ریسک فاکتور ( QEC) استفاده شد. یافته‌ها: پرسش‌نامه نوردیک در بین 216 نفر از پرستاران توزیع گردید. تعداد 29 نفر که دارای اختلال‌هاي عضلانی– اسکلتی بودند. میزان شیوع اختلال‌هاي عضلانی- اسکلتی در ناحیه کمر 4/64 درصد، زانو 4/51 درصد، گردن 6/48 درصد، شانه 5/43 درصد و مچ ‌دست 7/40 درصد، نواحی پشت 6/29 درصد، پا و قوزک پا 29 درصد، باسن– ران 5/24 درصد و آرنج 3/ 15 درصد بود. جا به جا کردن بیمار، بالاترین امتیاز را در میان وظایف به خود اختصاص داده است. به دنبال 8 هفته تمرین‌هاي اصلاحی و مداخله‌هاي ارگونومی میزان اختلال‌هاي کمر، شانه، گردن و مچ دست بهبودی معنی‌داری را نشان دادند (P<0/05). نتيجه‌گيري: میزان شیوع اختلال‌هاي عضلانی- اسکلتی در پرستاران مورد مطالعه به طور نسبي بالا بود. بالا بودن سطح ریسک خطر نشانه آسیب‌زا بودن شرایط و محیط کار در بیمارستان است. اصلاح تکنیک جا به جا کردن بیماران و بهبود شرایط بلند کردن بیماران توصیه می‌شود.

Title: کارآیی آموزش تغذیه بر اساس مدل بزنف در بهبود شاخص‌های متابولیکی سالمندان مبتلا به دیابت نوع 2

http://hsr.mui.ac.ir/index.php/jhsr/article/view/102

مقدمه: دیابت یکی از شایع‌ترین بیماری‌های مزمن می‌باشد امروزه دیابت در سالمندان یکی از مهم‌ترین اپیدمی‌های قرن است این مطالعه با هدف بررسی اثر بخشی مدل بزنف (باور‌ها، نگرش‌ها، نرم‌های انتزاعی و عوامل قادر‌کننده) در آموزش تغذیه کوتاه مدت به سالمندان (≥60 سال) مبتلا به دیابت نوع 2 انجام پذیرفت. روش‌ها: در این کار آزمایی بالینی تصادفی، 100 بیمار سالمند مبتلا به دیابت نوع 2 در دو گروه مداخله و کنترل به صورت تصادفی بر‌اساس شماره پرونده تقسیم شدند اطلاعات هر دو گروه به صورت مشابه و در دو زمان قبل و 3 ماه پس از مداخله آموزشی جمع‌آوری گردید. مداخله آموزشی در 4 جلسه آموزشی و هر جلسه به مدت 70 دقیقه انجام و نتایج به وسیله آزمون‌های آماری مجذور کای، T مستقل، زوجی و آنالیز واریانس مورد تجزیه و تحلیل قرار گرفت. یافته‌ها: پس از مداخله آموزشی میانگین کلیه سازه‌های مدل بزنف (آگاهی، نگرش، نرم‌های انتزاعی، عوامل قادر‌کننده) تفاوت معنی‌داری‌ را در بین‌ گروه مداخله و گروه کنترل نشان می‌دهد (001/0>P). علاوه بر آن میانگین هموگلوبین گلیکوزیله (001/0>P)، قند خون ناشتا (001/0>P)، تری گلیسیرید (001/0>P) و لیپوپروتئین سبک (02/0=P) بهبود معنی ‌‌‎داری را در گروه مداخله نشان می‌دهد. نتیجه‌گیری: مداخله آموزش تغذیه بر اساس مدل بزنف علاوه بر بهبود آگاهی و نگرش سالمندان مبتلا به دیابت نوع 2 سبب بهبود شاخص‌های متابولیک آن‌ها نیز شده است.

Title: بررسی شیوع چاقی و اضافه وزن در جانبازان شهر اصفهان در سال 1388

http://hsr.mui.ac.ir/index.php/jhsr/article/view/154

مقدمه: برخی مطالعات انجام شده ارتباط بين چاقي واضافه وزن با نمایه توده بدنی وتوده چربي را درافراد معلول نشان داده‌اند. با توجه به اين‌كه چاقي واضافه وزن عوارض جدي برروي سلامتي دارد، در اين مطالعه بر آن شديم تا ميزان شيوع چاقي واضافه وزن را در گروهي از جانبازان بررسي كنيم. روش‌ها: درمطالعه مقطعی حاضر تعداد 106 نفر از جانبازان شهراصفهان مورد مطالعه قرارگرفتند. جمع‌آوری داده‌ها شامل داده‌های تن سنجی وتاریخچه پزشکی انجام و داده‌ها در نرم‌افزار 10SPSS ثبت گردید. یافته‌ها: میانگین وانحراف معیار شاخص‌های تن سنجی به شرح زیر می‌باشد: وزن Kg 44/13 ± 75/78، قد cm 04/8 ± 39/168، نمایه توده بدنی  Kg/m268/4 ± 98/27، دور وسط بازو cm 12/4 ± 19/33، دور ساق پا cm 38/7 ± 96/31، محیط دور عضله بازو cm 02/11 ± 34/27، چین پوستی دو سر بازو mm 47/5 ± 10/12، چین پوستی سه سر بازو mm 08/6 ± 35/16، چین پوستی زیر کتفی mm 10/8 ± 17/30، چین پوستی بالای ایلیاک mm 33/7 ± 82/32، چربی کل mm 16/24 ± 13/89. نتیجه‌گیری: با توجه به يافته‌ها بیش از نیمی از جمعیت مورد مطالعه دچاراضافه وزن و چاقی هستند. اين در حالي است كه جانبازان قطع نخاعي با نمایه توده بدنی یكسان در مقایسه با ديگر جانبازان، ميزان توده چربي بيشتري داشتند که ازعلل عمده آن می‌توان به کم تحرکی در این گروه اشاره کرد. البته بررسی بیشتر و با حجم نمونه بیشترتوصیه می‌شود.

Title: بررسی وضعیت بهداشت محیطی رستوران‌ها و هتل‌های دارای سالن غذاخوری در محدوده گردشگری بخش مرکزی شهر اصفهان با استفاده از GIS در سال 87 و 88

http://hsr.mui.ac.ir/index.php/jhsr/article/view/108

مقدمه: بهداشت مواد غذایی و محل‌های توزیع مواد غذایی دارای اهمیت فراوان می‌باشد. گردشگران به جایی سفر می‌کنند که از اعتماد، اطمینان و امنیت کافی برخوردار باشد. در این شرایط ارایه خدمات و امکانات مناسب نظیر محل‌های توزیع مواد غذایی با شرایط بهینه بهداشتی در افزایش انگیزه آن‌ها مؤثر است. هدف پژوهش حاضر، بررسی وضعیت بهداشت محیطی رستوران‌ها و هتل‌های دارای سالن غذا‌خوری مرکز توریستی شهر اصفهان بود، که سالانه پذیرای مسافران فراوانی است. روش‌ها: این تحقیق به صورت توصیفی و نمونه‌گیری به صورت غیر احتمالی هدف‌دار انجام شد. اطلاعات بازرسی بهداشت محیطی از رستوران‌ها و هتل‌های دارای سالن غذا‌خوری مراکز بهداشتی و درمانی 16 ملک و خواجو در طی سال‌های 87 و 88 جمع‌آوری و خلاصه گردید، سپس توسط نرم‌افزار 15SPSS و 2007 Excel مورد آنالیز توصیفی و تحلیلی قرار گرفت. تصویر اصفهان با استفاده از نرم‌افزارهای Google earth و Corel draw تهیه شده، در نرم‌افزار 0/2 Arc view با توجه به اطلاعات بهداشت محیط مورد تجزیه و تحلیل قرار گرفت. یافته‌ها: مراکز بهداشتی و درمانی 16 ملک و خواجو در مجموع دارای  26 واحد بودند، که 17 مورد تحت پوشش مرکز 16 ملک (65 درصد کل واحدها) و 9 مورد تحت پوشش مرکز خواجو (35 درصد کل واحدها) قرار داشت. 65 درصد این واحدها رستوران و 35 درصد نیز هتل‌های دارای سالن غذاخوری بودند. میانگین تعداد بازدید از هر واحد در طی 2 سال در کل منطقه و در مراکز 16 ملک و خواجو به ترتیب برابر 41/11، 07/12 و 25/10 بازدید بود. این میانگین در سال اول و دوم به ترتیب برابر 6 و 32/5 بازدید و برای رستوران و هتل‌ها به ترتیب برابر 77/11 و 89/10 بازدید به دست آمد. نتیجه‌گیری: وضعیت بهداشت محیط در سال 87 و همچنین در منطقه خواجو و در هتل‌ها به ترتیب از وضعیت بهداشت محیط در سال 88، منطقه 16 ملک و رستوران‌ها بهتر بود. وضعیت بهداشت محیط در دو واحد معادل 9 درصد در سطح یک (خوب)، 10 واحد معادل 45 درصد در سطح دو (خوب)، 8 واحد معادل 36 درصد در سطح سه (متوسط) و 2 واحد معادل 9 درصد در سطح چهار (ضعیف) قرار داشتند.
